# Supplementary material for: Altered Transcriptional Regulation of Glycolysis in Circulating CD8+ T Cells of Rheumatoid Arthritis Patients
Source: Genes (Basel). 2022 Jul 7;13(7):1216. doi: 10.3390/genes13071216 (PMC9323564; doi:10.3390/genes13071216)
Supplement: Supplementary file 1 [file genes-13-01216-s001.zip › genes-1694431-supplementary figures S1-S12.pdf]

Supplementary Figures

Comparison of untreated RA and TCZ treated CD8$^+$ Tem cells

The expression levels of genes from glycolysis, pentose phosphate pathway, TCA cycle, Oxidative phosphorylation complex 1, 4 and 5 in the CD8$^+$ Tem cells from healthy, untreated and TCZ treated individuals are shown in the following figures. Significant differential expression is marked by asterisk (***).

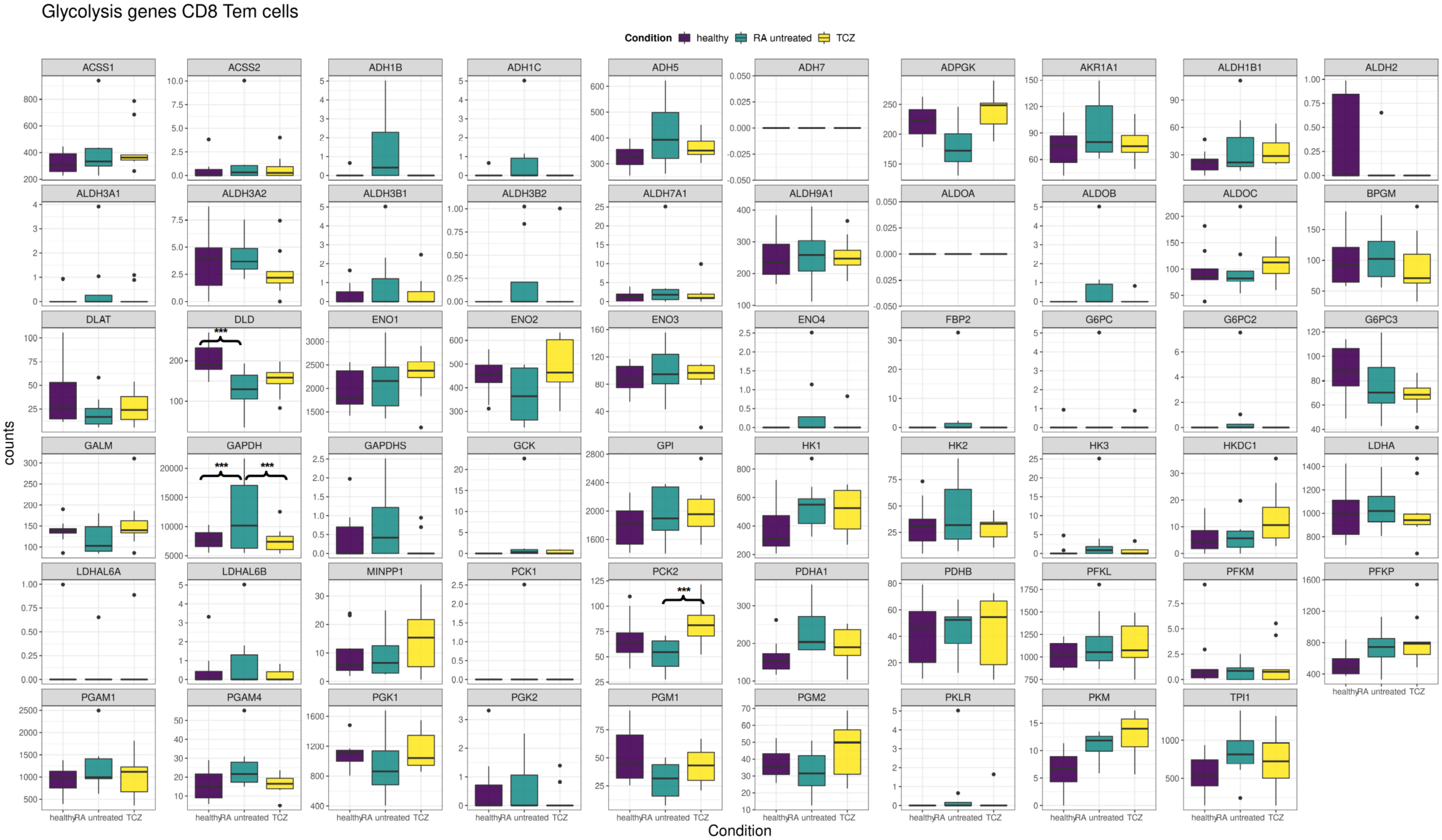

Figure S1. Normalized counts of glycolysis genes in untreated RA CD8$^+$ Tem cells and TCZ treated RA CD8$^+$ Tem cells.

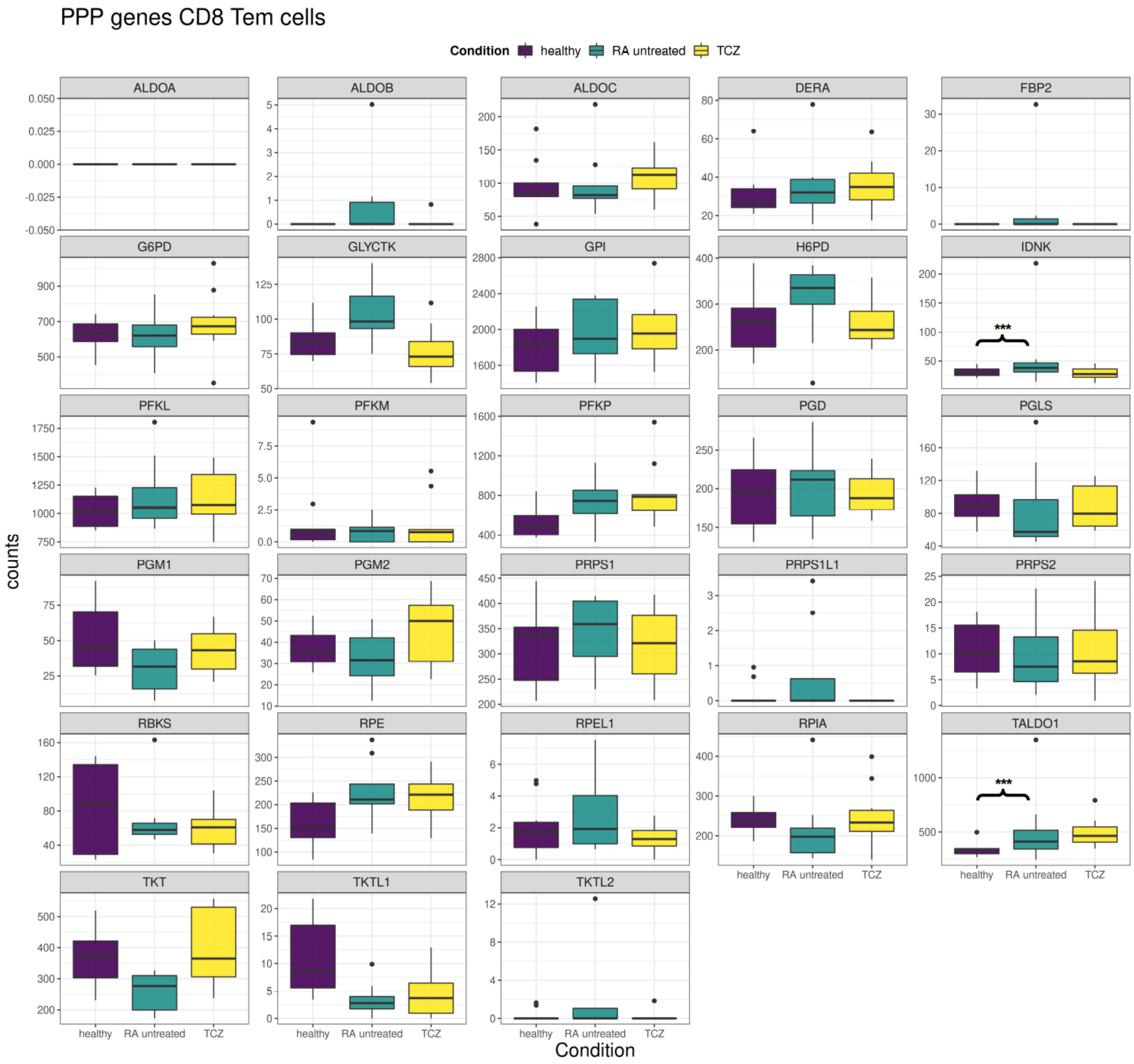

Figure S2. Normalized counts of pentose phosphate pathway genes in untreated RA CD8⁺ Tem cells and TCZ treated RA CD8⁺ Tem cells.

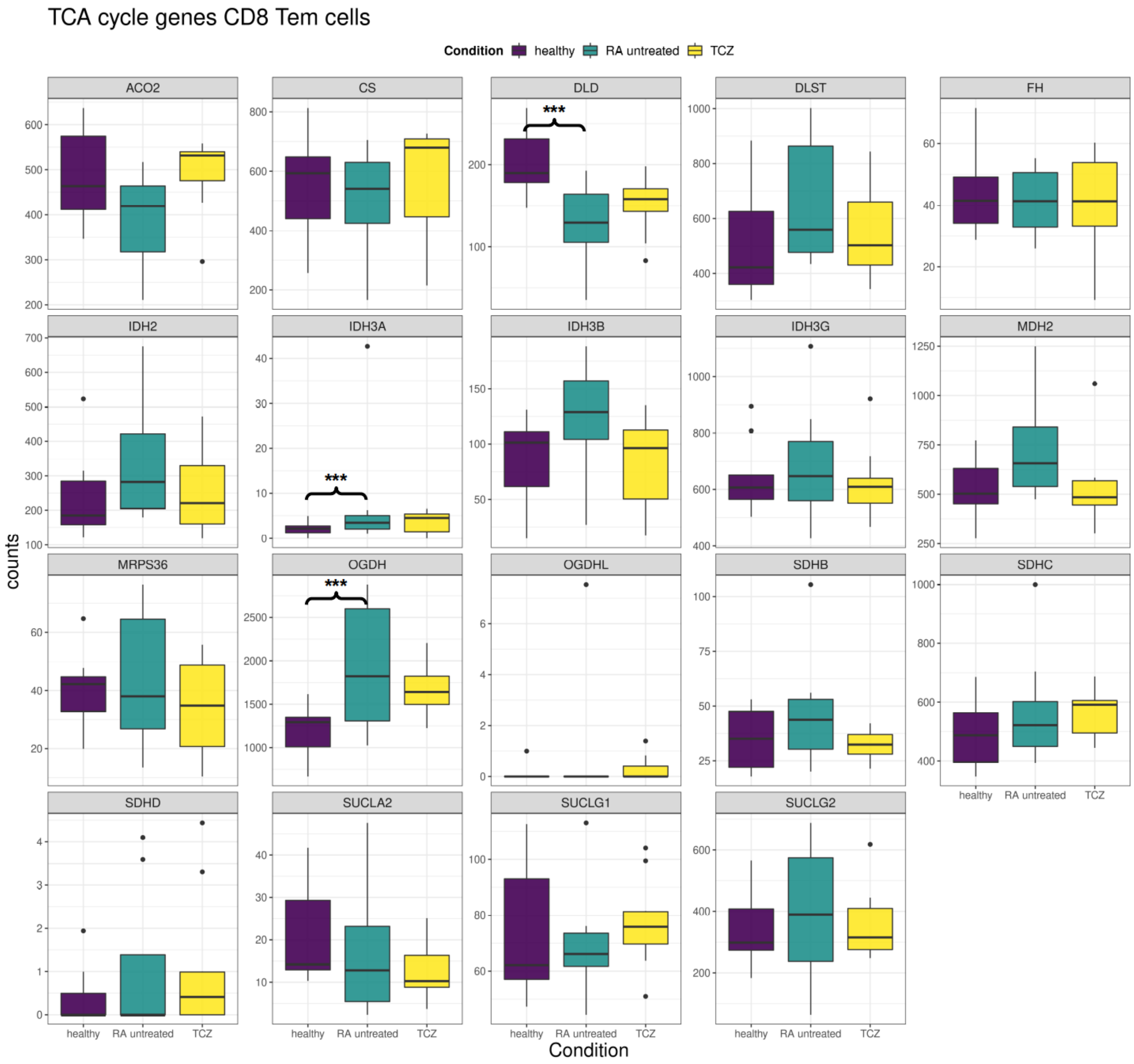

Figure S3. Normalized counts of TCA cycle genes in untreated RA CD8^{+} Tem cells and TCZ treated RA CD8^{+} Tem cells.

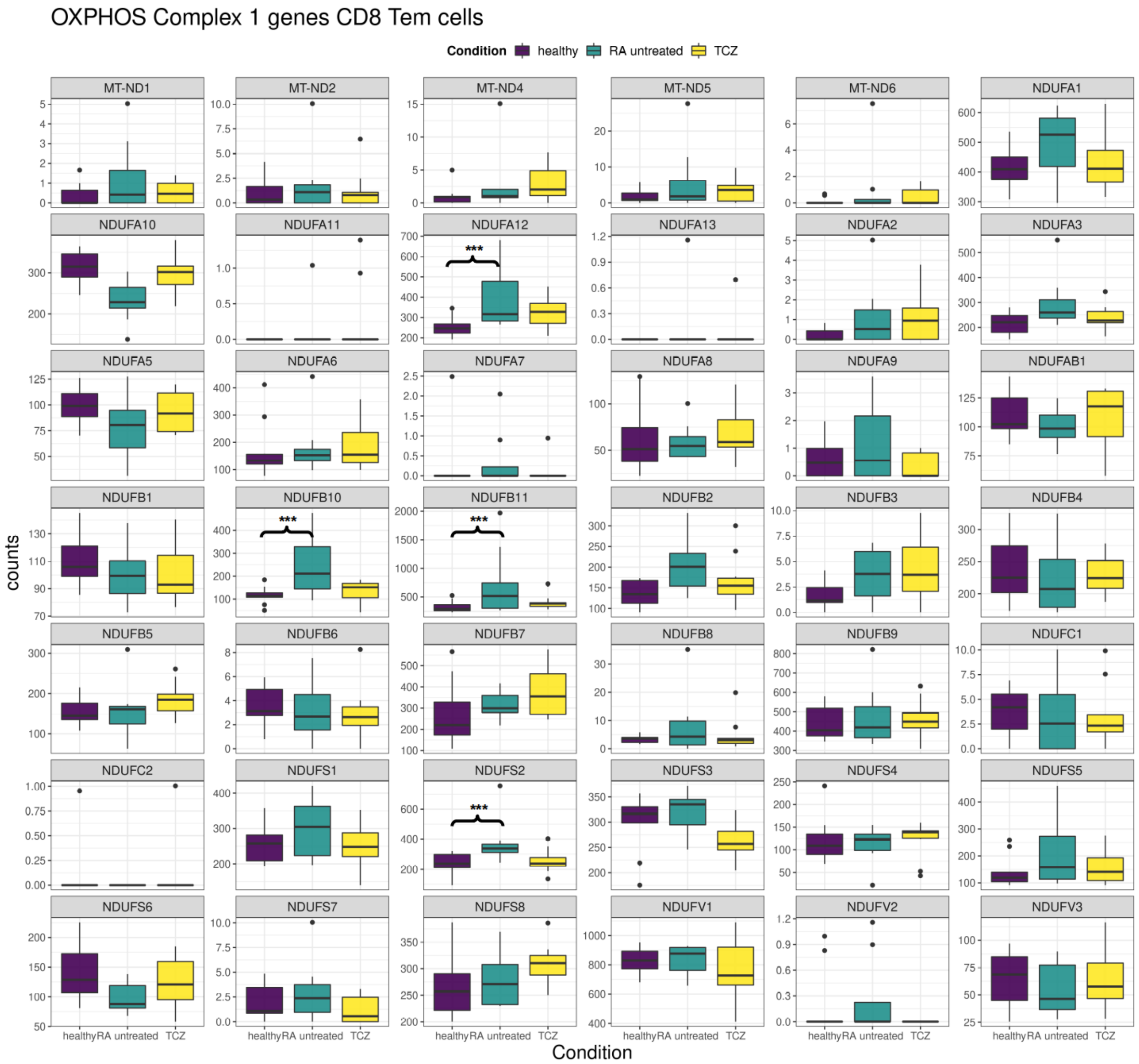

Figure S4. Normalized counts of oxidative phosphorylation complex 1 genes in untreated RA CD8⁺ Tem cells and TCZ treated RA CD8⁺ Tem cells.

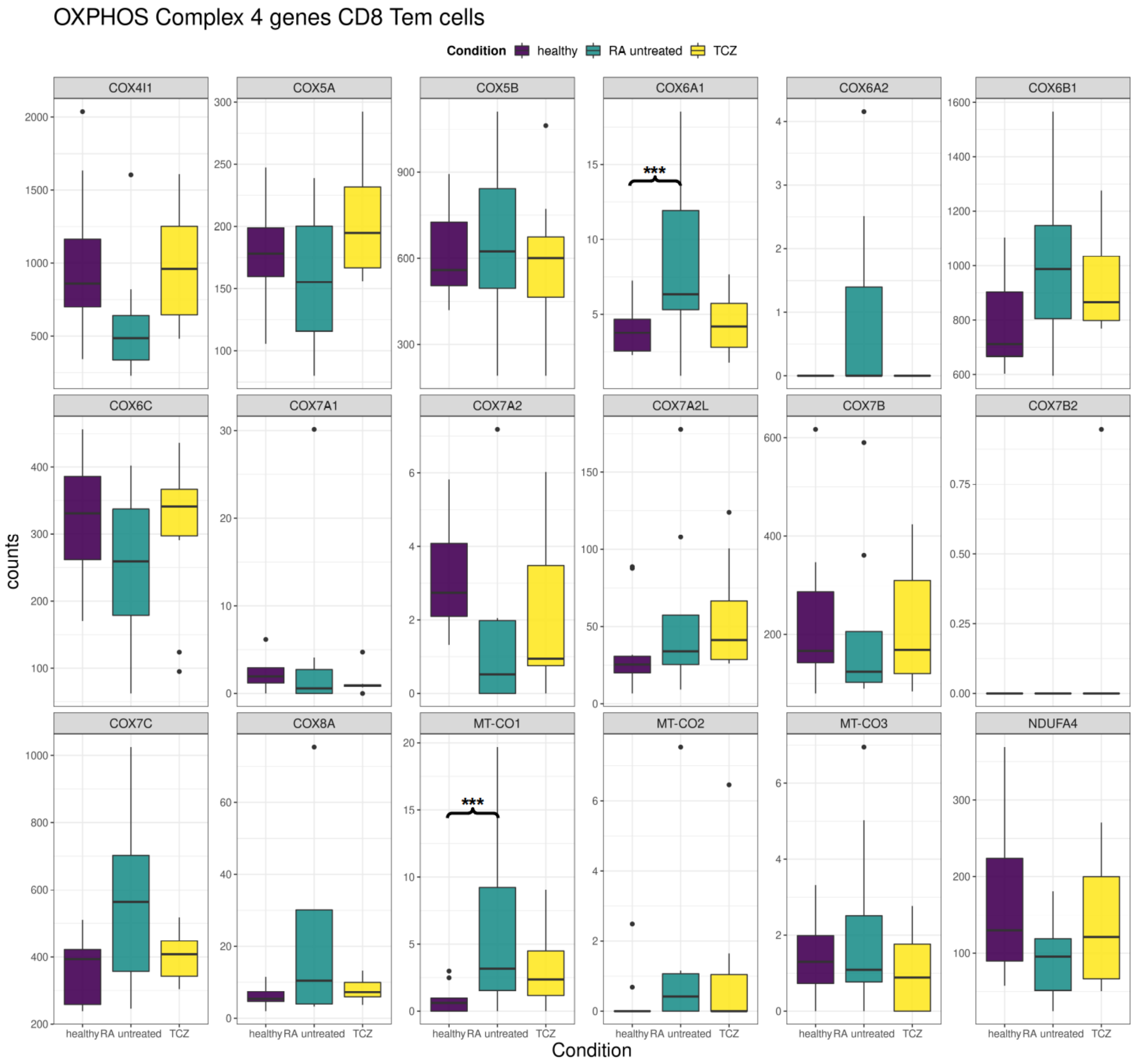

Figure S5. Normalized counts of oxidative phosphorylation complex 4 genes in untreated RA CD8+ Tem cells and TCZ treated RA CD8+ Tem cells.

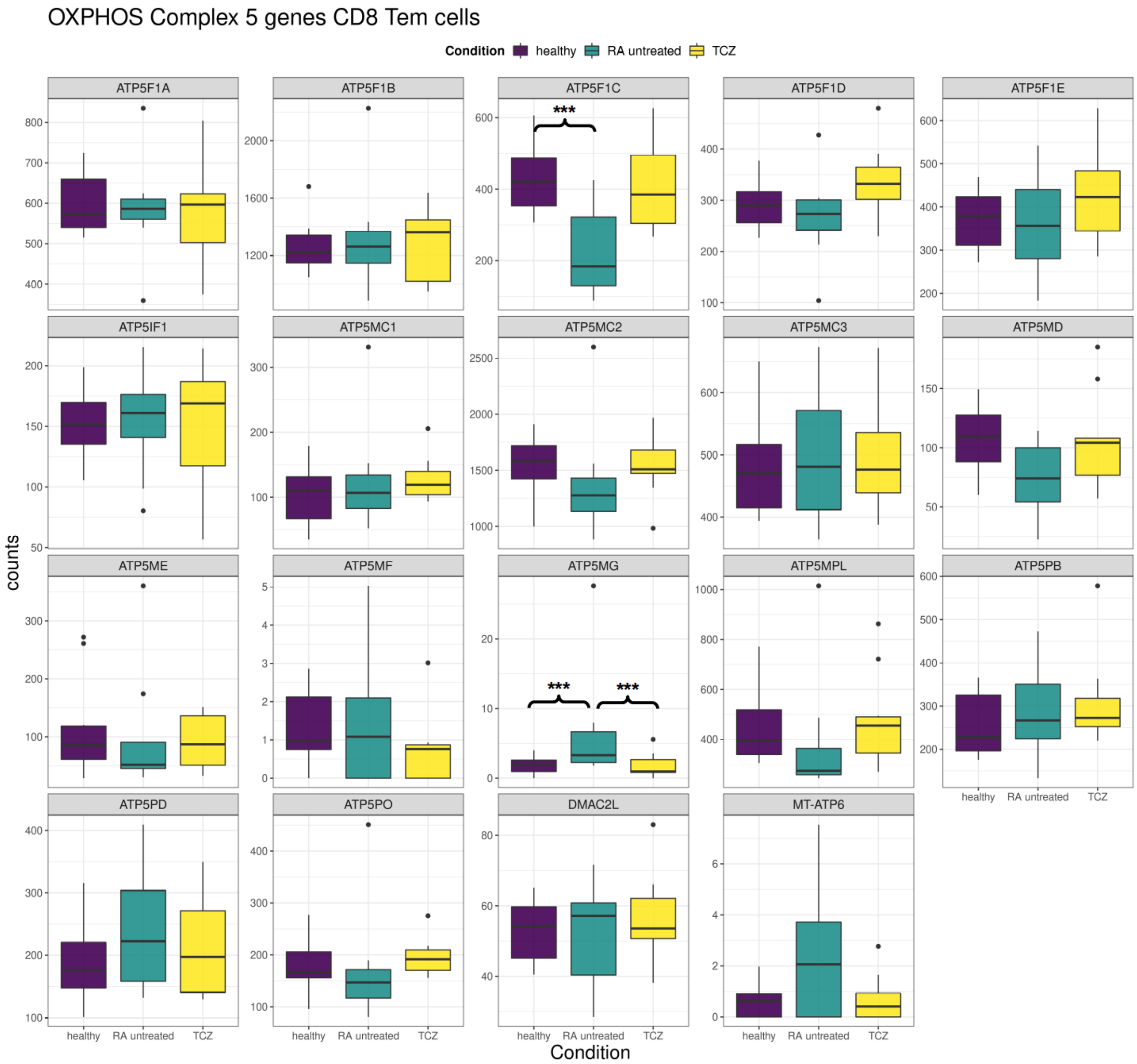

Figure S6. Normalized counts of oxidative phosphorylation complex 5 genes in untreated RA CD8⁺ Tem cells and TCZ treated RA CD8⁺ Tem cells.

Comparison of untreated RA CD8⁺ Tem cells and CD8⁺ Temra cells

The expression levels of genes from glycolysis, pentose phosphate pathway, TCA cycle, Oxidative phosphorylation complex 1, 4 and 5 in the CD8⁺ Tem and CD8⁺ Temra cells from healthy and untreated RA individuals are shown in the following figures. Significant differential expression is marked by asterisk (***).

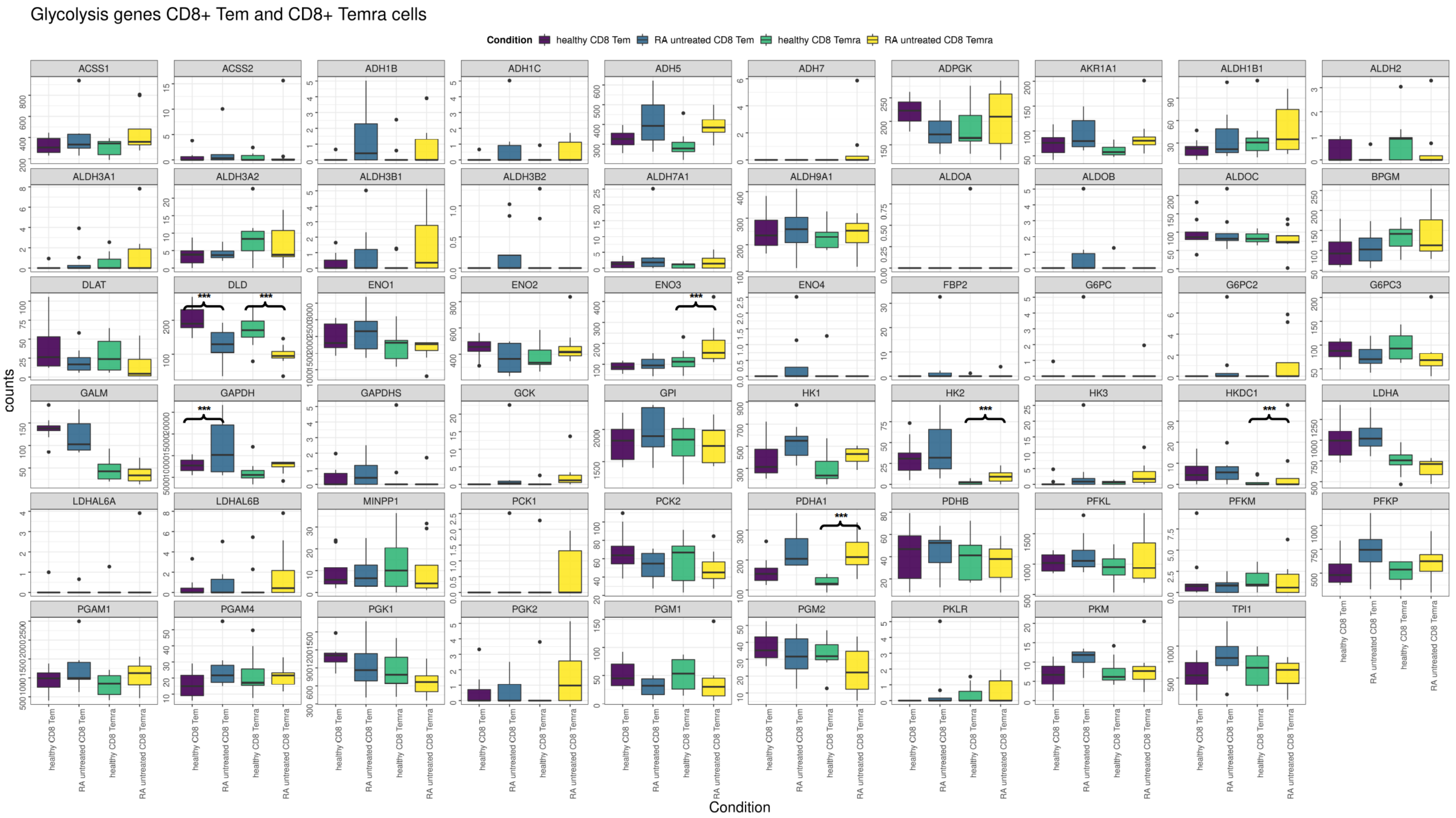

Figure S7. Normalized counts of glycolysis genes in untreated RA CD8^{+} Tem cells or in untreated RA CD8^{+} Temra cells

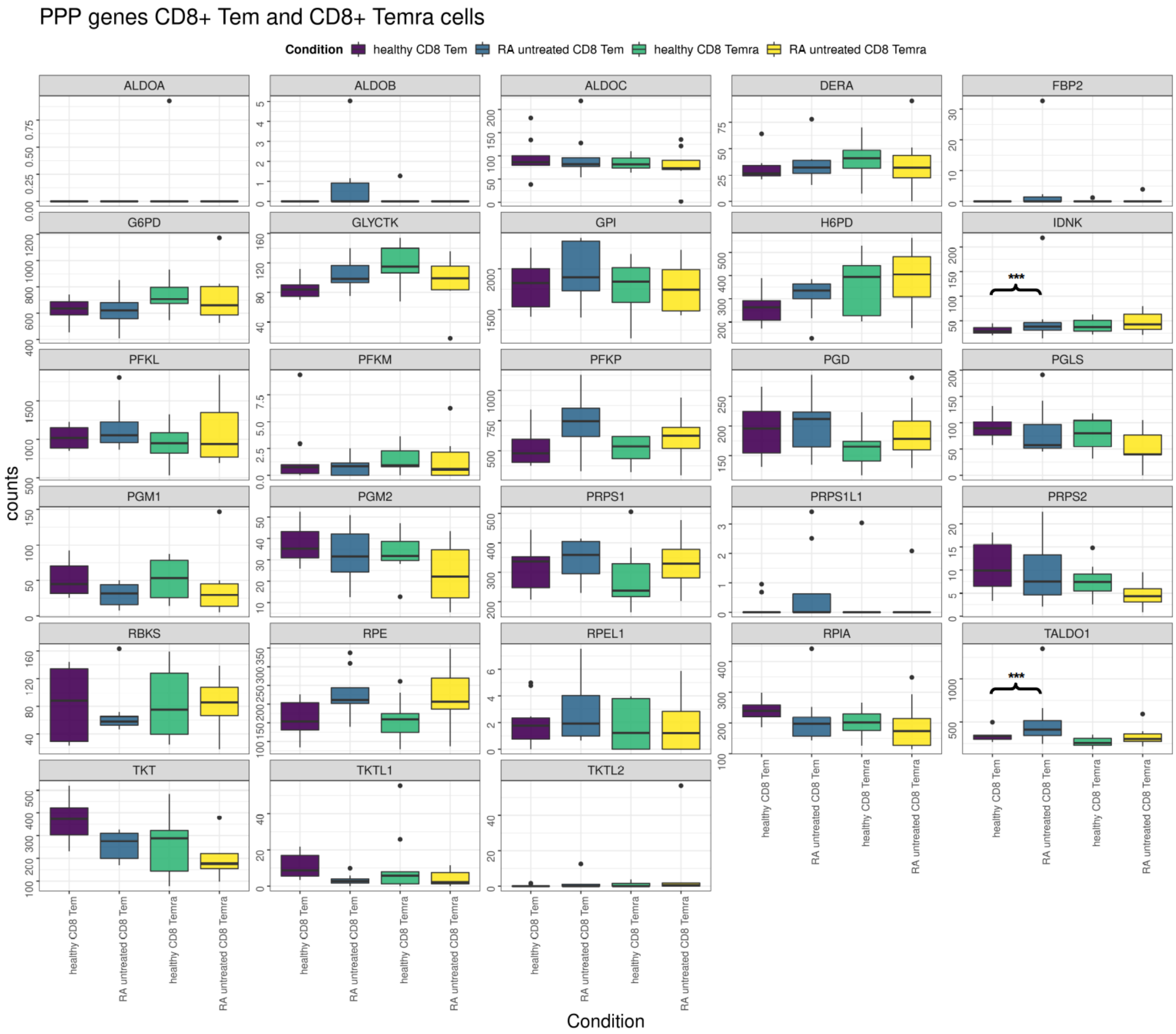

Figure S8. Normalized counts of pentose phosphate pathway genes in untreated RA CD8+ Tem cells or in untreated RA CD8+ Temra cells

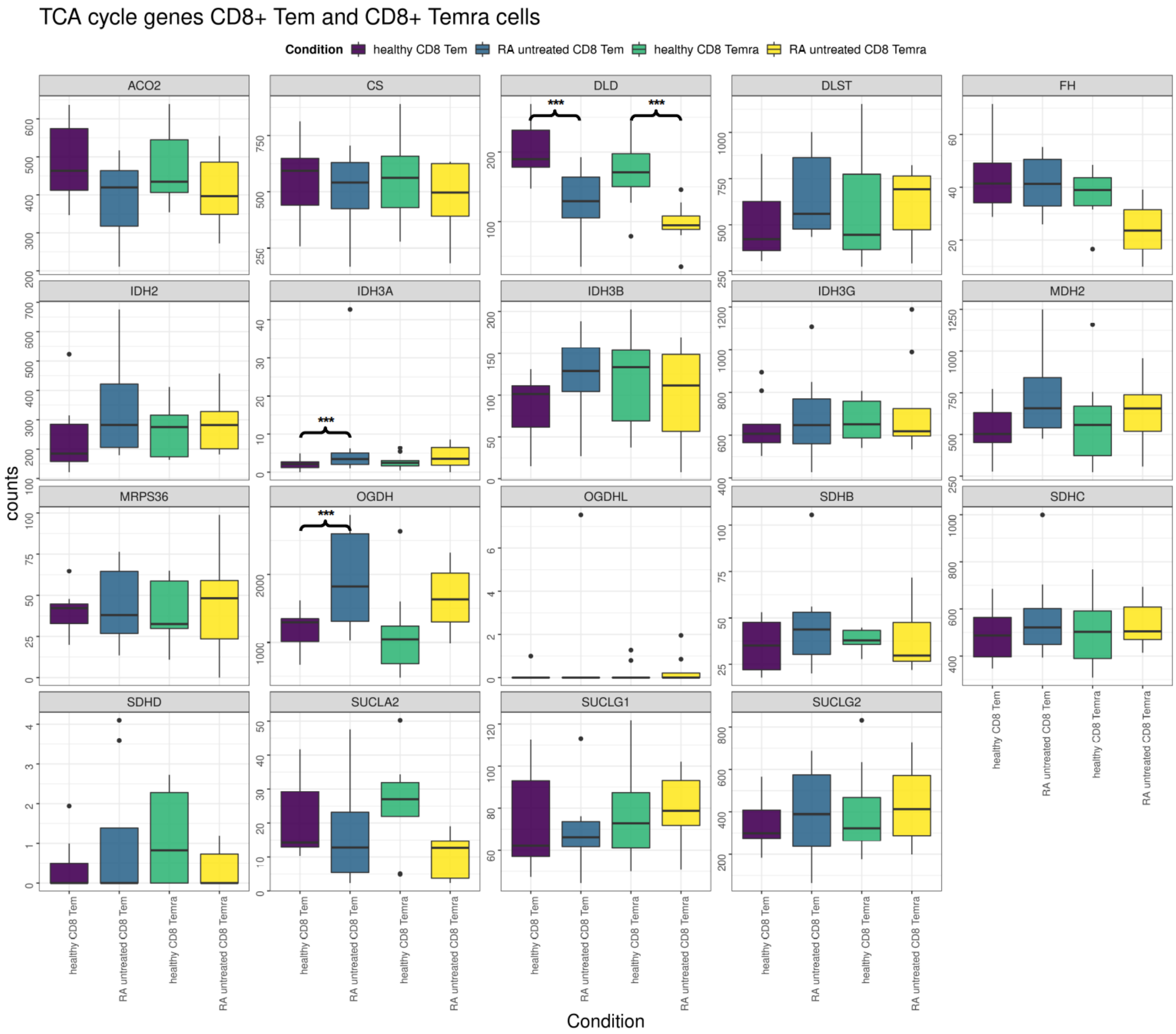

Figure S9. Normalized counts of TCA cycle genes in untreated RA CD8^{+} Tem cells or in untreated RA CD8^{+} Temra cells

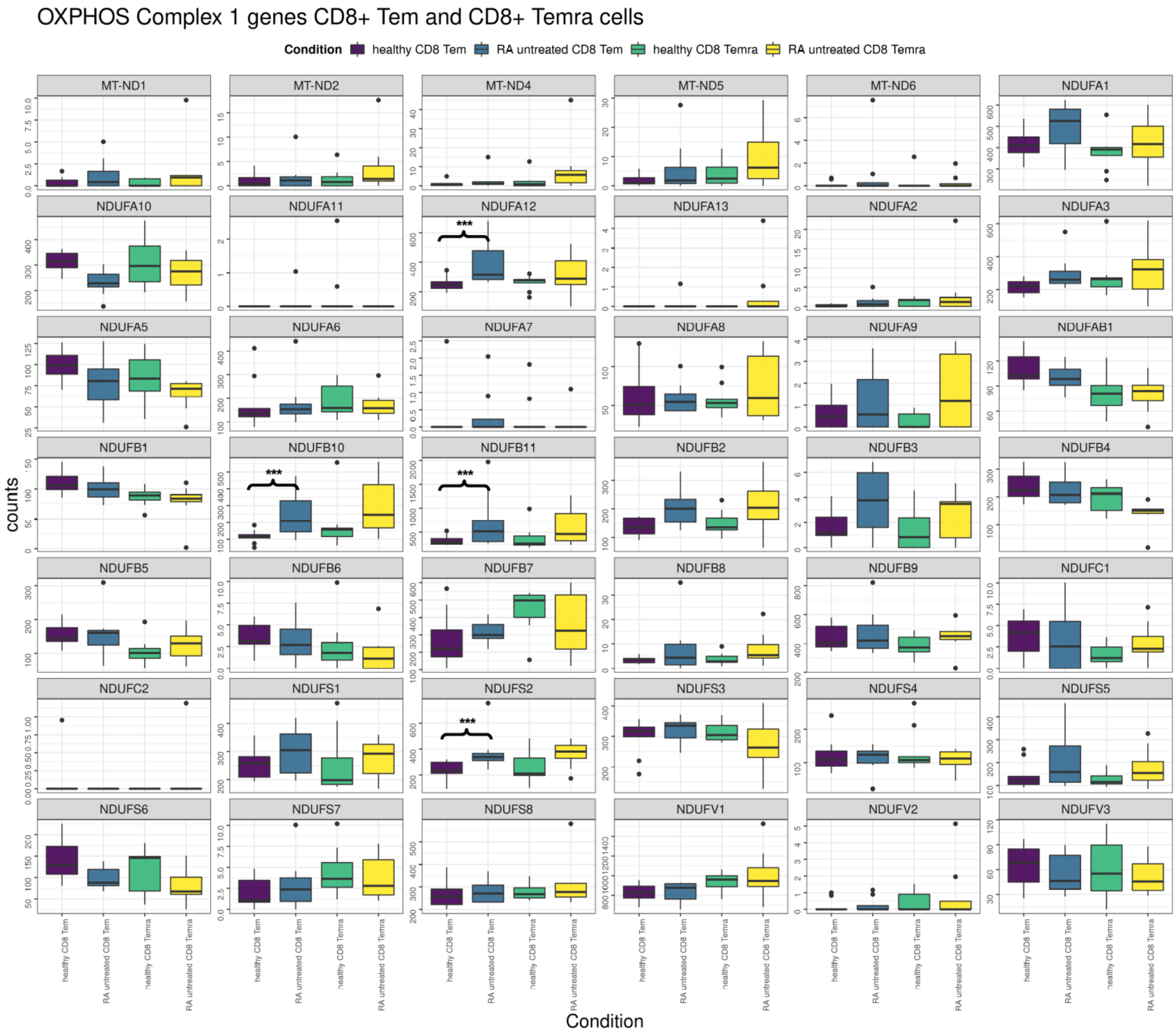

Figure S10. Normalized counts of oxidative phosphorylation complex 1 genes in untreated RA CD8^{+} Tem cells or in untreated RA CD8^{+} Temra cells

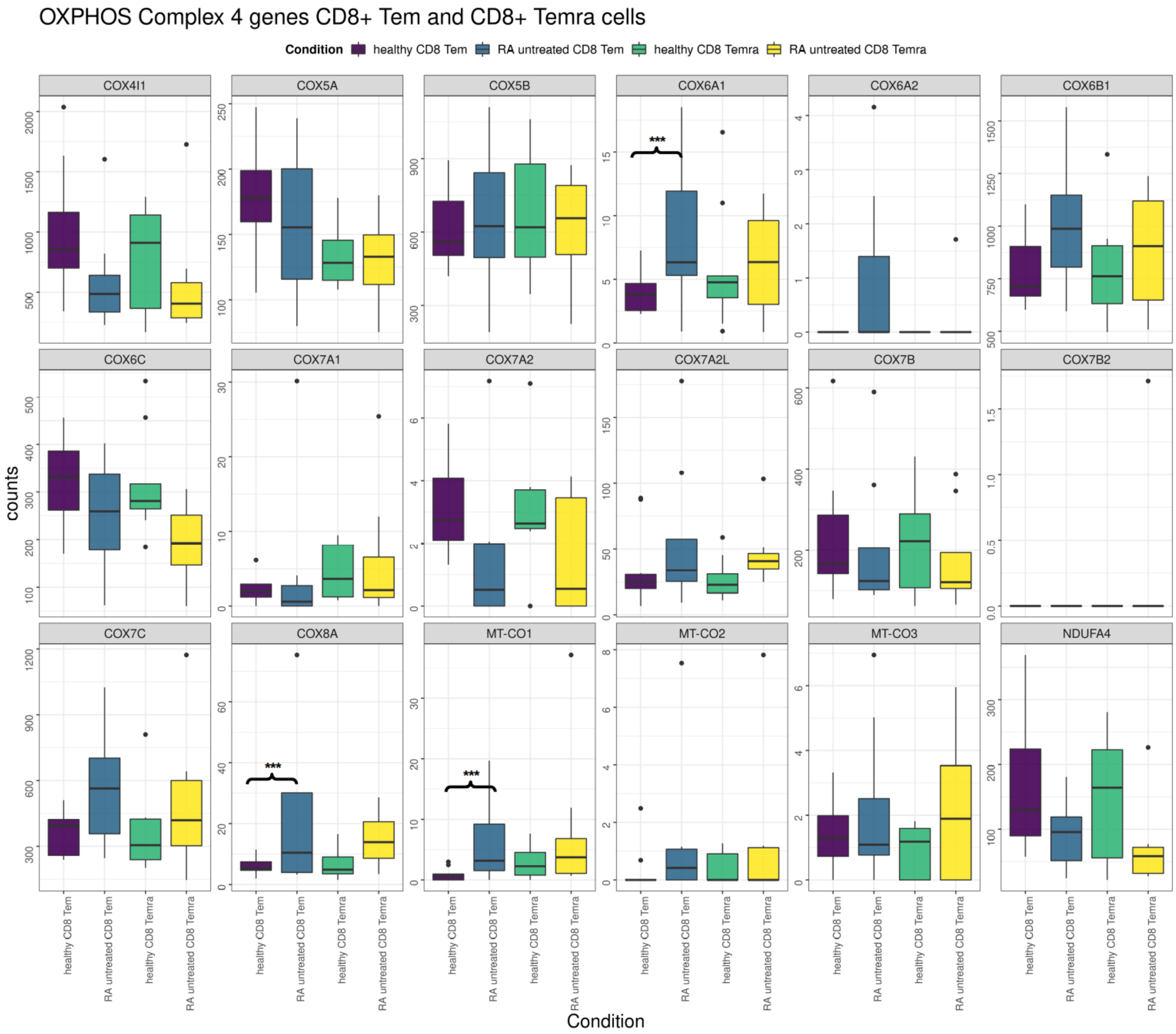

Figure S11. Normalized counts of oxidative phosphorylation complex 4 genes in untreated RA CD8+ Tem cells or in untreated RA CD8+ Temra cells

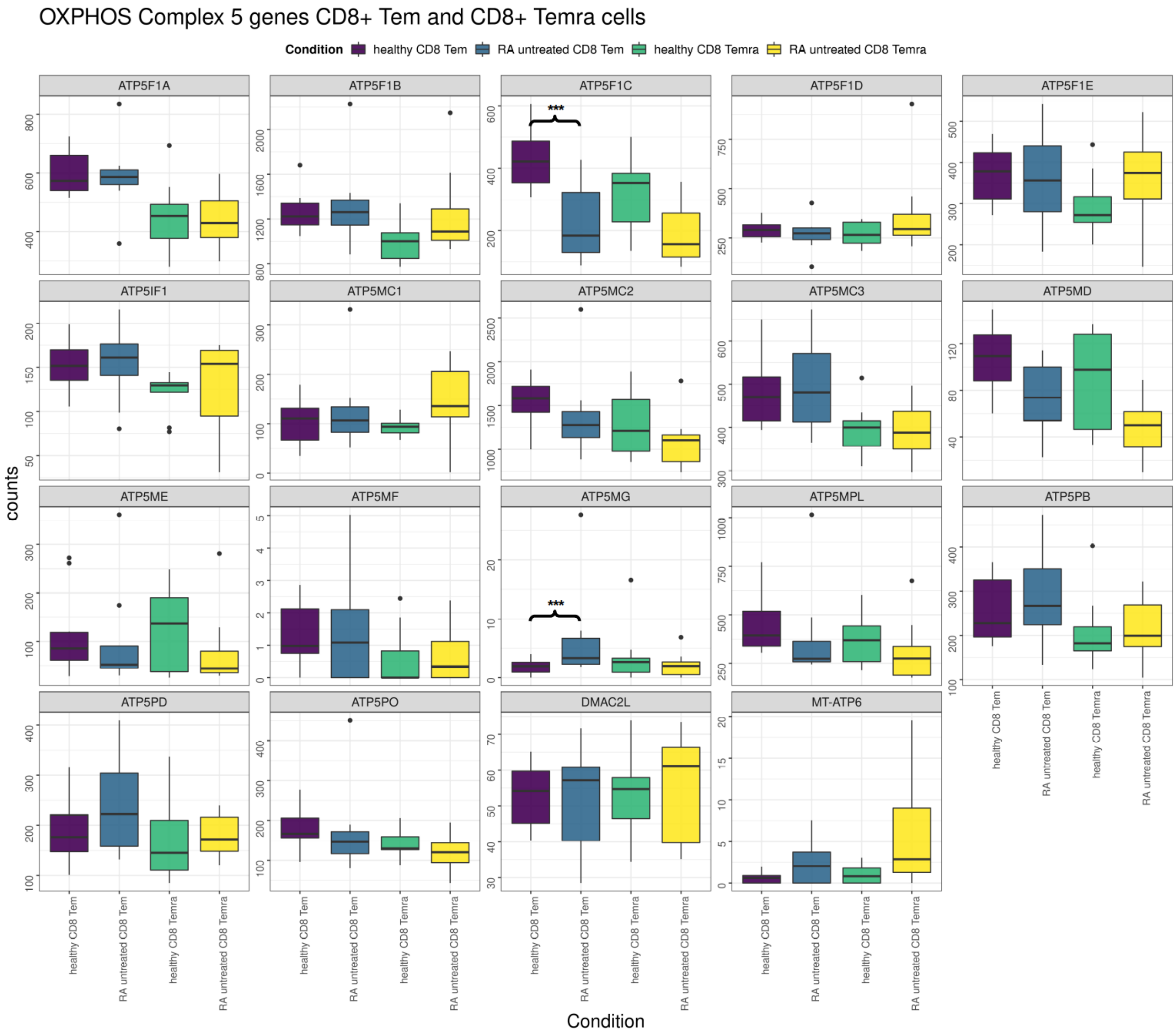

Figure S12. Normalized counts of oxidative phosphorylation complex 45 genes in untreated RA CD8^{+} Tem cells or in untreated RA CD8^{+} Temra cells
